# Supplementary material for: Autologous Peripheral Blood Mononuclear Cells in Patients with Small Artery Disease and Diabetic Foot Ulcers: Efficacy, Safety, and Economic Evaluation
Source: J Clin Med. 2023 Jun 20;12(12):4148. doi: 10.3390/jcm12124148 (PMC10298945; doi:10.3390/jcm12124148)
Supplement: Supplementary file 1 [file jcm-12-04148-s001.zip › jcm-2421855-supplementary.pdf]

SUPPLEMENTARY MATERIALS

Table S1– Costs (EUR) for hospital admission for foot-related conditions.

DRG code and description of procedure	Hospital length >1 day	Day-hospital
114 – Toe amputation for vascular diseases	8,962	731
205 – Lower limb amputation for metabolic or endocrinological disease vascular diseases	13,431	482
130 – Peripheral revascularization with multiple comorbid conditions	4,904	390
131 – Peripheral revascularization without multiple comorbid conditions	3,398	390
556 – Peripheral revascularization with drug-eluting stent	10,097	731
418 - Post-surgical infection	3,862	453
238 – Osteomyelitis<br>575 or 576 – Sepsis	5,974<br>6,974	379<br>453

§ Hospital length without any additional costs; *additional daily costs for hospitalization > length threshold

Table S2 – Costs (EUR) associated to procedures and laboratory examinations.

**DRG code and description of procedures**	**Costs** (€)
88.28 Foot/ankle X-ray	21
88.38.7 Foot/ankle computed tomography	173
88.77.2 Lower limbs ecocholor doppler	49.5
88.94.1 Foot/ankle Nuclear Magnetic Resonance	254
89.65.4 Transcutaneous oxygen monitoring	18.6
90.16.3 Creatinine	1.2
90.28.1 Glycated Hemoglobin	10.6
90.27.1 Glycemia	1.30
90.14.3 Total cholesterol	1.1
90.14.1 HDL cholesterol	1.8
90.43.2 Triglycerides	1.3
90.72.3 C-reactive protein	3.6
86.11 Cutaneous biopsy	13.9
90.85.2 Swab with antibiogram	12.2
99.24.2 Antibiotic infusion (cost for antibioitics not included)	3.1
